# Supplementary figures and images for: Predicting disease occurrence of cabbage Verticillium wilt in monoculture using species distribution modeling
Source: PeerJ. 2020 Nov 17;8:e10290. doi: 10.7717/peerj.10290 (PMC7678443; doi:10.7717/peerj.10290)

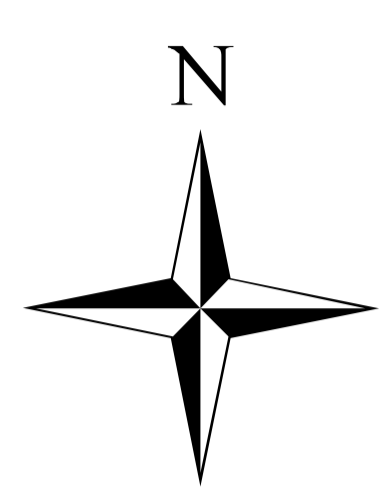

138°27'0"E

138°32'30"E

36°35'30"N

36°30'0"N

36°24'30"N

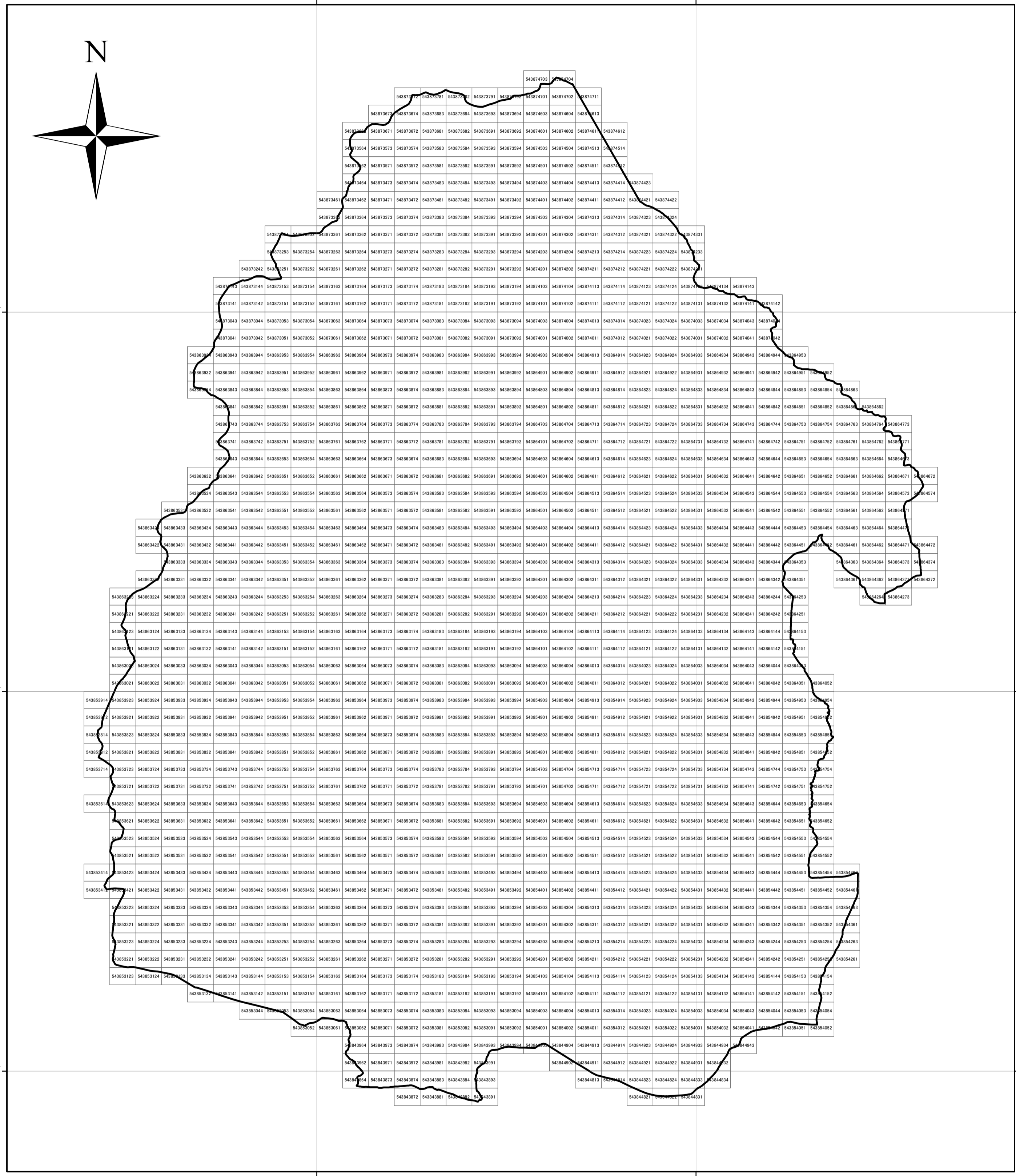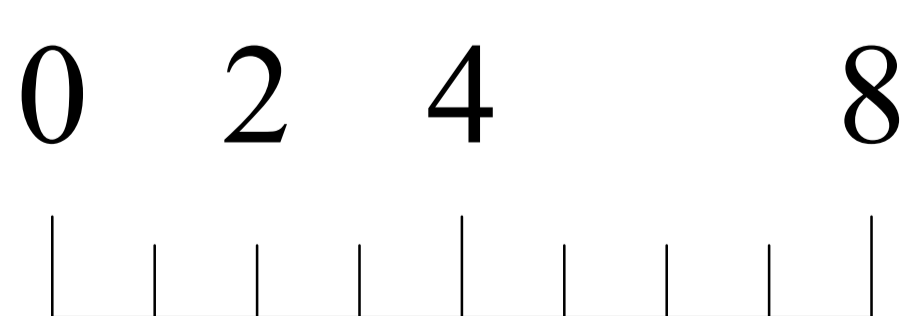

km

Supplement: Supplemental Information 3 — The study area was divided into a grid of 500 × 500 m cells. [file peerj-08-10290-s003.pdf]
